# Identification and environment-friendly biocontrol potential of five different bacteria against *Aphis punicae* and *Aphis illinoisensis* (Hemiptera: Aphididae)

**DOI:** 10.3389/fmicb.2022.961349

**Published:** 2022-10-25

**Authors:** Alaa Baazeem, Saqer S. Alotaibi, Luaay Kahtan Khalaf, Uttam Kumar, Madiha Zaynab, Sarah Alharthi, Hadeer Darwish, Akram Alghamdi, Suresh Kumar Jat, Amal Al-Barty, Bander Albogami, Ahmed Noureldeen, Balasubramani Ravindran

**Affiliations:** ^1^Department of Biology, College of Science, Taif University, Taif, Saudi Arabia; ^2^Department of Biotechnology, College of Science, Taif University, Taif, Saudi Arabia; ^3^Department of Plant Protection, College of Agricultural Engineering Science, University of Baghdad, Baghdad, Iraq; ^4^College of Plant Protection, Fujian Agriculture and Forestry University, Fuzhou, China; ^5^Shenzhen Key Laboratory of Marine Bioresource and Eco-environmental Sciences, College of Life Sciences and Oceanography, Shenzhen University, Shenzhen, Guangdong, China; ^6^Department of Chemistry, College of Science, Taif University, Taif, Saudi Arabia; ^7^Department of Plant Protection, College of Horticulture and Forestry, Agriculture University, Kota, India; ^8^Department of Environmental Energy and Engineering, Kyonggi University, Suwon, South Korea; ^9^Department of Medical Biotechnology and Integrative Physiology, Institute of Biotechnology, Saveetha School of Engineering, Saveetha Institute of Medical and Technical Sciences, Thandalam, Chennai, Tamil Nadu, India

**Keywords:** entomopathogenic bacteria, molecular identification, *Aphis punicae*, *Aphis illinoisensis*, biological control

## Abstract

The current work is aimed at isolating and identifying new Entomopathogenic bacterium (EPB) strains associated with *Steinernema feltiae* and assessing the EPB’s biocontrol potential on *Aphis punicae* and *Aphis illinoisensis* adults in the laboratory. From *S. feltiae*, five bacterial isolates were isolated and molecularly characterized. *Lysinibacillus xylanilyticus* strain TU-2, *Lysinibacillus xylanilyticus* strain BN-13, *Serratia liquefaciens* strain TU-6, *Stenotrophomonas tumulicola* strain T5916-2-1b, and *Pseudochrobactrum saccharolyticum* strain CCUG are the strains. Pathogenicity tests demonstrated that bacterial cells were more toxic against the two aphid species than bacterial cell-free supernatants. *S. tumulicola* strain T5916-2-1b cells and filtrate were reported to have the strongest potential to kill *A. punicae* and *A. illinoisensis* individuals within 6 h after treatment, with 100% mortality of both insects 24 and 48 h after treatment. Based on the results of the study, it looked like endogenous *Steinernema*-associated EPB could be used directly as a biocontrol agent for *A. punicae* and *A. illinoisensis*.

## Introduction

Since several years, entomopathogenic nematodes (EPNs) from the Steinernematidae and Heterorhabditidae families have been investigated as biocontrol agents. This is because they have a wide range of hosts and host-seeking behavior, as well as mass rearing and are easily applicable with low cost. They also exhibit long-term efficacy, they are compatible with most chemicals, they are safe for the environment (for non-target organisms such as humans), their use reduces the amount of pesticide residues in food, they increase the activity of other natural enemies and they increase biodiversity in managed ecosystems (Gaugler and Kaya, 1990).

Gram-negative bacteria from the Enterobacteriaceae family, *Photorhabdus* sp. and *Xenorhabdus* sp., live in symbiotic associations with EPNs from the *Heterorhabditis* and *Steinernema* genera, respectively (Boemare et al., 1993; Sajnaga and Kazimierczak, 2020). Infective juveniles (IJs) of steinernematid and heterorhabditid nematodes carry the symbiotic bacteria in their midguts, which inhabit in the soil of various ecological systems (Dillman et al., 2012). The nematodes aggressively seek for insect hosts, the symbiotic *Xenorhabdus* sp. and *Photorhabdus* sp. are released into the hemocoel after entering by the insect’s mouth, anus, or spiracles, respectively (Salvadori et al., 2012). The symbiotic bacteria play a wide range of biological roles, the most important of which is to keep the pathobiome conditions in the polyxenic colonized insect cadaver and soil appropriately balanced for the EPN/EPB symbiotic complex (Ogier et al., 2020). The symbiotic bacteria then invade the insect’s haemolymph, destroy tissues, and explore a variety of immunosuppressive factors, such as toxin complexes, hydrolytic enzymes, hemolysins, and antimicrobial substances that kill the insect host in less than 48 h (Fang et al., 2011; Shi et al., 2017). Finally, in the insect host, the symbiotic bacteria multiply fast, causing septicaemia a process for turning insect cadavers into a suitable food source for nematode development and reproduction. Multiple recent investigations, however, when entomopathogenic partners were injected into insects alone, the results put a question mark on this hypothesis, they were found to exhibit decreased virulence or to be nonvirulent (Bisch et al., 2015; Kim et al., 2017; McMullen et al., 2017). Similarly (Ogier et al., 2020), revealed that the association between *Steinernema* and *Xenorhabdus* was not monoxenic, and that several Proteobacteria were found in the bacterial population associated with laboratory-reared IJs from *Steinernema carpocapsae*, *S. feltiae*, *S. glaseri*, and *S. weiseri*. They also observed that a dozen Proteobacteria species (*Pseudomonas*, *Stenotrophomonas*, *Alcaligenes*, *Achromobacter*, *Pseudochrobactrum*, *Ochrobactrum*, *Brevundimonas*, *Deftia*, and others) were found to be linked with the main symbiont (*Xenorhabdus nematophila*). Non-symbiotic bacteria are hypothesized to join IJ vectors *via* the cuticle or intercuticular region, where they eventually enter the insect haemocoel during IJ penetration.(Singh et al., 2014). In soil-dwelling *Caenorhabditis elegans* nematodes*, Pseudomonas*, *Ochrobactrum*, and *Stenotrophomonas* have been frequently identified (Dirksen et al., 2016). Likely, Proteobacteria are also the most common bacterial group found in plant root bacterial populations.(Hartman et al., 2017) and in soils covered with plants, such as the rhizosphere (Kumar et al., 2022).

Many insect pests affect the quality and yield of pomegranate and grapevine cultivation. The pomegranate aphid, *Aphis punicae* Passerini (Hemiptera: Aphididae), is a major pest attacks pomegranate crop around the world. Fruits, leaves, and inflorescences are consumed by both adults and nymphs. Pomegranate aphid infestation results in pale, curled leaves, slowed development, and dropped flowers, as well as the transmission of viral infections and the secretion of honey dew, which fungi survive in lowering crop quality and yield (Moawad and Al-Barty, 2011). *Aphis illinoisensis* (Shimer) is a grapevine pest that feeds on the lower surface of new leaves, young terminal shoots (Blackman and Eastop, 2011), and fruit clusters, causing some grape berries to fall off (Pfeiffer and Schultz, 1986). Pomegranate and grapevine aphids have been documented as invasive pests in southern European, North African, and Asian countries since the early 2000s (El-Gantiry et al., 2012; Salim et al., 2022). Unfortunately, aphids have a high reproductive potential, and using insecticides extensively to control them leads to resistance development. When aphicides are used heavily on pomegranates or grapevines, the remnants are mainly concentrated in the fruits. Contamination with pesticides is undesirable because these fruits are consumed fresh (Li and Han, 2004; Pertot et al., 2017). As a result, scientists are looking for new pesticides that are more efficient against pests, less hazardous to natural enemies, and less destructive to the environment (Fouad et al., 2018; Ga’al et al., 2021).

Root weevils, white grubs, root worms, cutworms, sciarid flies, and armyworms are among the pests that have been controlled by EPNs (Hazir et al., 2004). When tested in field and laboratory conditions, EPNs and/or entomopathogenic bacteria (EPB) have been shown to satisfactorily control mosquitoes, pomegranate aphids, cabbageworms, scarab beetles and cherry fruit flies (Herz et al., 2006; Alghamdi et al., 2017; Yooyangket et al., 2018; Elbrense et al., 2021). The gene encoding the protease inhibitor protein has been recognized and expressed in the symbiotic bacterium *Xenorhabdus b*ovienii strains BJFS526 and Xbpi-1. This protein’s impact on the pea aphid *Acyrthosiphon pisum* was also investigated (Zeng et al., 2012; Jin et al., 2014). *Xenorhabdus szentirmaii* is a one-of-a-kind source of antimicrobial peptides that are effective against virtually all known phytopathogens (Fuchs et al., 2014; Fodor et al., 2022).

To date, in several countries, including Saudi Arabia, both EPNs and their associations have not been sufficiently examined in terms of their diversity and application. Considering all the plant protection perspectives, as well as climatic, geographic and regulation aspects, the most reasonable approach is to search for potential biological plant protection (EPN/EPB) agents native locally.

Therefore, various bacterial strains that could be used as suitable control organisms need to be assessed to develop a new biological control technique. The genetic diversity of Saudi Arabian and Egyptian EPN genotypes was examined using RAPD and ISSR markers after an EPN species, *Steinernema* sp., was isolated from the soil of pomegranate trees in Taif, Saudi Arabia (Aljuboori et al., 2022). Geographically, Taif is an elevated location in Saudi Arabia, with valleys, steep mountains, and agricultural plateaus. There is an abundance of potential insect hosts; thus, the diversity of EPNs and EPB is expected to be very rich in this region. The goals of this research was to identify EPB associated with *Steinernema* found in Taif, Saudi Arabia, and to assess their activity against the pomegranate and grapevine aphids, *A. punicae* and *A. illinoisensis*, under laboratory conditions. On the basis of these goals we hypothesized that EPB associated with *Steinernema* would be good controlling agents for the control of *A. punicae* and *A. illinoisensis*.

## Materials and methods

### Insects

Fresh leaves and buds of pomegranate and grapevine trees infested with *A. punicae* and *A. illinoisensis*, respectively, were harvested on the same experimental day from pomegranate and grapevine farms in Taif, Saudi Arabia.

### Isolation of *Steinernema*-associated bacteria

In this study, EPN *S. feltiae* strain NYH (MTöth et al., 2005) was originated from the Laboratory of Fodor Andras, Pannonia University, Keszthely, Hungary. According to the Akhurst (1980) method modified by (Vitta et al., 2018), bacterial symbionts of *S. feltiae* were isolated from infective dauer juveniles (IJs) or from the haemolymph of deceased *Galleria mellonella* larvae that was infected with *S. feltiae* IJs. Briefly, to isolate EPB from EPN infective juveniles, IJs were collected and centrifuged three times using sterilized tap water after being obtained from Galleria white traps. Some were placed in sterile petri plates with a drop of physiological saline (M9) solution before being moved to 5% chlorox. Individuals were moved to a series of sterilized M9 drops after a 2-min incubation period, and then fractured with a sterile platinum wire. The drop of M9 was diluted and deposited onto an NBTA indicator plate [nutrient agar + triphenyl tetrazolium chloride (0.004%) and bromothymol blue (0.025%)] and incubated at 28°C for 48 h. For isolation of EPB from *G. mellonella* cadavers, the dead *G. mellonella* larvae were washed in 100% ethanol for 1 min to be surface-sterilized before being placed in a sterile Petri dish to dry. Following that, a sterile sharp needle was used to penetrate the third segment of the *G. mellonella* larvae’s head to allow an influx of the haemolymph containing symbiotic bacteria. The haemolymph samples were distributed and streaked over NBTA media using a sterile loop, as previously described. Bacteria were frequently cultured every 24 h until pure isolated colonies were acquired, and then stored at −80°C with 20% glycerol (*v*/*v*) for further study. To generate the cell-free conditioned filtrates or cell suspensions, in 5 ml of Luria-Bertani (LB) broth, one colony of every isolate of relevant bacteria was seeded and cultured overnight at 28°C shaking at 220 rpm. Furthermore, 100-ml culture aliquots were shaken at room temperature overnight before being introduced to flasks with 400 ml of the identical media and agitated at 200 rpm for 5 days. To obtain a cell-free filtrate, the supernatant was filtered by a 0.22 μm Millipore filter, then the pellet was resuspended in sterile distilled water. Following that the filtrate was kept at 4°C for subsequent dilution with sterile distilled water to get concentrations of 600, 400, 200, and 100 μl/ml. The bacterial cell suspension was adjusted at OD_600_ to 1.0 using a spectrophotometer. A 10-fold serial dilution spread plate was used, with a bacterial suspension concentration of 1 × 10^8^ (CFU/ml). Each bacterial cell solution was diluted to obtain concentrations 10^8^, 10^6^, 10^4^, and 10^2^ CFU/ml.

### Identification of *Steinernema*-associated bacteria

The genomic DNA of the isolated bacteria was extracted from the bacterial pellets using the Bacterial Genomic DNA Miniprep Kit (QIAprep Spin Miniprep Kit). The bacterial genomic DNA was stored at −20°C prior to use in a PCR. To identify bacterial species, PCR-based analysis and *16S rRNA* gene sequencing (1,504 base pairs, bp) were completed using the inter-universal primers 785F (GGATTAGATACCCTGGTA) and 907R (CCGTCAATTCMTTTRAGTTT; Tailliez et al., 2006).

### Phylogenetic tree analysis

To identify the bacterial species associated with *S. feltiae*, a comparison of the partially edited nucleotide sequences (16S rRNA) was performed using the BLASTN program from the NCBI. The 16S rRNA sequences were compared to the NCBI database “16S rRNA sequence (Bacteria and Archae).” The alignments of all 16S sequences were done operating the program of MUSCLE with 50 iterations and were presented in the CLC viewer. The evolutionary history was inferred using the most probability method supported the Tamura-Nei model (Tamura and Nei, 1993). The tree with the highest log likelihood (−1426.34) is shown. The proportion of trees within which the associated taxa clustered together is shown next to the branches. Initial tree(s) for the heuristic search were obtained automatically by applying Neighbor-Joining and BioNJ algorithms to a matrix of pairwise distances estimated using the most composite likelihood (MCL) approach and so selecting the topology with superior log likelihood value. Evolutionary analyses were performed employing MEGA7 (Kumar et al., 2018) with 1,000 bootstrap values.

### Bioassay

The five *Steinernema*-associated bacterial isolates (cells or filtrates) were selected for use in the toxicity bioassay: *Stenotrophomonas tumulicola* strain T5916-2-1b (Isolate 7), *Pseudochrobactrum saccharolyticum* strain CCUG (Isolate 13), *Lysinibacillus xylanilyticus* strain BN-13 (Isolate 1), *Serratia liquefaciens* strain TU-6 (Isolate 6) and *Lysinibacillus xylanilyticus* strain TU-2 (Isolate 2). Accession numbers have been given in Table 1. The toxicity of the bacterial isolates was evaluated on *A. punicae* and *A. illinoisensis via* a topical application method described by Eidy et al. (2016), with slight modification. In brief, five Petri dishes (9 cm) lined with filter paper (Whatman number 2) were prepared for each bacterial cell or filtrate concentration, and then four Taify pomegranate or grapevine leaf discs with a diameter of 1.5 cm were cut out and placed on the filter paper in each dish to feed the aphids. Then, 5 μl of each concentration of bacterial cell suspension or supernatant was dropped directly onto the bodies of the aphids. The individual adult aphids were carefully relocated using a fine camel hairbrush onto the leaf discs in the Petri dishes. In the control conditions, insects were treated with the same volume of distilled water or sterile filtered LB media for each aphid species. After that, the Petri dishes were wrapped with Parafilm and held under laboratory conditions of 25 ± 1°C, 65 ± 3% relative humidity and a 12 l:12 D light–dark cycle. The mortality rate of the aphids was recorded after exposure to the bacterial suspensions or cells for 6, 12, 24 and 48 h. If an insect’s appendages did not move when pushed with a fine-point brush, it was declared dead. Each bioassay was performed with five replicates on different dates. The experiment was repeated twice. Furthermore, LC_50_ and LC_90_ values of both the EPB cells and filtrates were determined using Probit analysis (Finney, 1971).

**Table 1:** Bacterial accession numbers.

Isolates	Bacterial strain	Accession number
Isolate 1	*Lysinibacillus xylanilyticus* strain BN-13	OP001649
Isolate 2	*Lysinibacillus xylanilyticus* TU-2	OP578131
Isolate 6	*Serratia liquefaciens* strain TU-6	OP578132
Isolate 7	*Stenotrophomonas tumulicola* strain T5916-2-1b	OP002058
Isolate 13	*Pseudochrobactrum saccharolyticum* strain CCUG 33852	OP002063

### Statistical analysis

A two-way variance analysis (ANOVA) was operated to evaluate the aphid mortality rate, followed by Duncan’s multiple range tests. The results were presented as mean ± standard error (*M* ± SE). The COSTAT program was used to conduct all analyses. (Version 6.400). Using SPSS Version 23, the values of LC_50_ and LC_90_, the 95% confidence limits of the lower and upper values, slope and intercept and the *χ*^2^ values of the tested EPB were *t*-tested (*p* < 0.05), where *p-*values less than 0.05 were significantly considered.

## Results

### Identification of EPBs by sequencing the 16S rRNA gene

A BLASTN search of the rRNA_type strains/16S_ribosomal_RNA database returned the following results. Isolate 1 showed 87.50% identity with *Lysinibacillus fusiformis* strain DSM 2898 (NR_042072.1) and with *Lysinibacillus fusiformis* strain NBRC 15717 (NR_112569.1). Similarly, Isolate 2 showed 88.15% identity with *Lysinibacillus fluoroglycofenilyticus* strain cmg86 (NR_148289.1) and with *Lysinibacillus sphaericus* strain DSM 28 (NR_042073.1), *Bacillus ndiopicus* strain FF3 (NR_149205.1), *Lysinibacillus macrolides* (NR_114920.1), *Solibacillus isronensis* (NR_115952.1) and *Lysinibacillus boronitolerans* (NR_041276.1). However, isolate 2 showed identity (98% similarity) with *Lysinibacillus xylanilyticus* strain TU-2. Isolate 6 showed 99.26% identity with *Serratia liquefaciens* strain ATCC 27592 (NR_122057.1), and Isolate 7 showed 99.56% identity with *Stenotrophomonas maltophilia* strain NBRC 14161 (NR_113648.1), *Stenotrophomonas tumulicola* strain T5916-2-1b (NR_148818.1) and *Stenotrophomonas pavanii* strain ICB 89 (NR_116793.1). Isolate 13 showed 88.26% identity with *Pseudochrobactrum saccharolyticum* (NR_042473.1).

**Figure 1:**
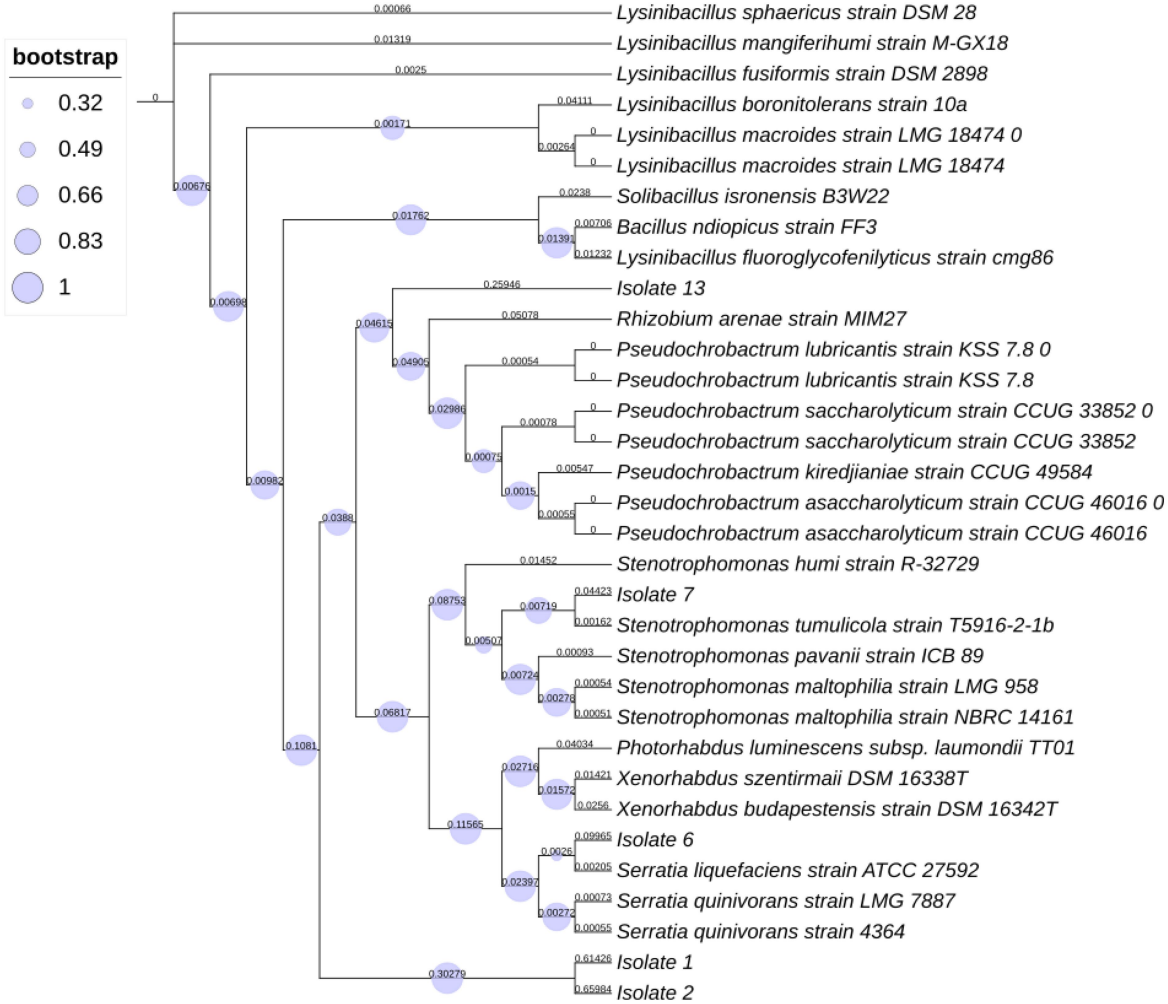
Phylogenetic tree represents distances based on the 16S rRNA sequences acquired from isolates within insect pathogenic nematodes *Steinernema feltiae*.

The phylogenetic tree analysis results (Figure 1) validated our morphological identification as indicated that Isolate 7 belonged to a *Stenotrophomonas tumulicola* T5916-2-1b and that Isolate 6 belonged a *Serratia liquefaciens* strain TU-6. However, isolate 1 belonged to *Lysinibacillus xylanilyticus* strain BN-13. Whereas, isolate 2 were found to be closely related to *Lysinibacillus xylanilyticus* strain TU-2. Additionally, isolate 13 was found to be closely related to *Pseudochrobactrum* sp.

### Insecticidal activity

#### Toxicity of bacterial cell suspensions to *Aphis illinoisensis* and *Aphis punicae*

The data presented in Table 2 show that the mortality rate of *A. illinoisensis* adults varied from 0 to 100% after topical application of five bacterial cell suspensions at four concentrations for four exposure times. These results confirmed that the efficiency of the bacterial isolates was directly associated with concentration (Table 2). The data show that cells from all of the bacterial isolates had a significant impact on the mortality rates of *A. illinoisensis* adults to some extent (*p* < 0.05), as they caused various levels of mortality in the grapevine aphid (*p* < 0.05). Table 2 also shows that the *A. illinoisensis* adults were susceptible (*p* < 0.05) to all of the bacterial isolates at all tested concentrations and exposure periods. Average mortality rates of 77 and 45.8% were recorded for *S. tumulicola strain* T5916-2-1b and *S. liquefaciens* strain TU-6, respectively. Bacterial cells of *Lysinibacillus xylanilyticus* strain BN-13 were less effective, inducing a mean adult mortality rate of 12.8% (*p* < 0.05). When individuals were exposed to 10^8^ CFU/ml of *S. tumulicola* for 24 and 48 h, the greatest *A. illinoisensis* mortality rate (100%) was detected. There was also a real correlation between mortality rate and exposure time (*p* < 0.05). As a result, the mortality rate increased dramatically as the exposure period increased., and an adult mortality rate of 44.2% was recorded 48 h post-exposure. High mortality rates were associated with high concentrations of *S. tumulicola* and *S. liquefaciens* at all tested times, and considerable mortality rates were associated with *L. xylanilyticus* strain TU-2. In contrast to the bacterial cells of *S. tumulicola* causing significant aphid mortality at all tested times when at a low concentration (10^2^ CFU/ml), the mortality rate associated with *L. xylanilyticus* strain BN-13. Was not significantly different from that of the control treatments (absence of bacteria-derived products), specifically with a 6-h exposure (0%).

**Table 2:** Toxicity of five bacterial species cells against the grapevine aphid, *Aphis illinoisensis* under laboratory condition.

Bacterial species	Concentration (CFU ml^−1^)	^a^Mortality %				Bacterial species means
		6 h	12 h	24 h	48 h	
*Stenotrophomonas tumulicola* (Isolate 7)	10^2^	^b^48 ± 4.9	60 ± 0	60 ± 0	68 ± 4.9	**77a**
	10^4^	56 ± 4	64 ± 4	72 ± 4.9	88 ± 4.9	
	10^6^	72 ± 4.9	80 ± 0	88 ± 4.9	96 ± 4	
	10^8^	84 ± 4	96 ± 4	100 ± 0	100 ± 0	
*Pseudochrobactrum saccharolyticum* (Isolate 13)	10^2^	12 ± 4.9	20 ± 0	32 ± 4.9	36 ± 4	**34.5d**
	10^4^	16 ± 4	32 ± 4.9	36 ± 4	40 ± 0	
	10^6^	20 ± 0	36 ± 4	40 ± 0	44 ± 4	
	10^8^	36 ± 4	44 ± 4	52 ± 4.9	56 ± 4	
*Lysinibacillus xylanilyticus* strain TU-2. (Isolate 2)	10^2^	8 ± 4.9	16 ± 7.5	20 ± 6.3	28 ± 4.9	**40.3c**
	10^4^	24 ± 4	32 ± 4.9	36 ± 4	36 ± 4	
	10^6^	40 ± 0	44 ± 4	56 ± 4	60 ± 0	
	10^8^	44 ± 4	52 ± 4.9	68 ± 4.9	80 ± 0	
*Serratia liquefaciens* (Isolate 6)	10^2^	24 ± 4	28 ± 4.9	32 ± 4.9	48 ± 4.9	**45.8b**
	10^4^	24 ± 4	36 ± 4	40 ± 0	48 ± 4.9	
	10^6^	28 ± 4.9	40 ± 6.3	48 ± 4.9	68 ± 4.9	
	10^8^	44 ± 4	56 ± 4	76 ± 4	92 ± 4.9	
*Lysinibacillus xylanilyticus* strain BN-13 (Isolate 1)	10^2^	0 ± 0	4 ± 4	4 ± 4	4 ± 4	**12.8e**
	10^4^	8 ± 4.9	8 ± 4.9	12 ± 4.9	12 ± 4.9	
	10^6^	8 ± 4.9	12 ± 4.9	16 ± 4	20 ± 0	
	10^8^	16 ± 4	16 ± 4	28 ± 4.9	36 ± 4	
Control		0 ± 0	0 ± 0	0 ± 0	0 ± 0	**0f**
Exposure time means		**25.5d**	**32.3c**	**38.2b**	**44.2a**	

a Each treatment was represented by five replicates, each with 20 adults insect.

b Numbers in each column indicates to mortality ± standard error.

Means with different letters within the same column or row differ significantly (*p* < 0.05 using Duncan’s multiple range test).

The data in Table 3 confirmed that the bacterial cells of *S. tumulicola* and *S. liquefaciens* were the most effective against *A. illinoisensis* 48 h after treatment, with LC_50_ values of 2.75 × 10 and 6.76 × 10^3^ CFU/ml and LC_90_ values of 8.13 × 10^3^ and 3.24 × 10^7^ CFU/ml, respectively. Table 3 also shows that the cells of *L. xylanilyticus* strain TU-2 were the third most virulent and that *Pseudochrobactrum saccharolyticum* strain CCUG and *L. xylanilyticus* strain BN-13 were the least efficient against *A. illinoisensis* after different exposure durations with LC_50_ values of 7.41 × 10^4^, 1.26 × 10^7^ and 4.37 × 10^9^ CFU/ml, respectively. For the *A. illinoisensis* population, the highest degree of homogeneity was found in *S. tumulicola* and *S. liquefaciens* with slope values of 2.95 and 2.33, respectively, and the other tested bacterial cell species exhibited low slope values, indicating heterogeneity in the aphid response to these bacterial isolates (Table 3).

**Table 3:** Aphicidal activity of five bacterial species cells against *A. illinoisensis* after 48 h of exposure.

Bacterial species	LC_50_ CFU ml^−1^ (95% LCL–UCL)	LC_90_ CFU ml^−1^ (95% LCL–UCL)	Slope ± SE	Intercept	*X* ^2^	*p*-Value
*S. tumulicola*	2.75 × 10 (1–1.8)^*^	8.13 × 10^3^ (3.4–4.7)	2.95 ± 0.43	−0.49	2.39	0
*P. saccharolyticum*	1.26 × 10^7^ (4.8–9.4)	2.75 × 10^15^ (12.96–17.3)	0.24 ± 0.03	−1.19	1.21	0.007
*L. xylanilyticus* strain TU-2	7.41 × 10^4^ (4.3–5.4)	1.35 × 10^10^ (9–11.9)	0.76 ± 0.28	−0.73	1.83	0
*S. liquefaciens*	6.76 × 10^3^ (1.1–4)	3.24 × 10^7^ (6.98–9)	2.33 ± 0.42	−0.64	2.40	0.001
*L. xylanilyticus* strain BN-13	4.37 × 10^9^ (8.5–11.7)	3.16 × 10^17^ (15.4–19.1)	0.23 ± 0.07	−2.55	1.08	0

* Figures in parenthesis are expressed as a power of 10.

LC_50_, lethal concentration that kills 50% of insects; LC_90_, lethal concentration that kills 90% of insects; LCL, lower confidence limit; UCL, upper confidence limit; *X*^2^, Chi-square value; SE, standard error; and *p*-value, probability.

The toxic activity data of five *Steinernema*-associated bacterial species against *A. punicae* under laboratory conditions are presented in Table 4. These EPB cells were found to have a significant effect on adult aphid mortality (*p* < 0.05). Adult lethality was significantly greater in the *S. tumulicola* isolate (82%) than in the other isolates. Individual mortality rates increased substantially as bacterial cell concentration and exposure time increased (*p* < 0.05). On aphid infection, there was a strong interaction between EPB species, bacterial cell concentration, and exposure period (*p* = 0.0113), whereas the interaction between bacterial cell concentration and exposure time was insignificant (*p* = 0.5263). The maximum mortality rate (100%) was observed with individuals exposed to 10^8^ CFU/ml of *S. tumulicola*, 6–48 h post treatment (compared to treatment with distilled water), and the lowest mortality rate (4%) was recorded with the adults exposed to 10^2^ CFU/ml of *L. xylanilyticus* strain BN-13, 6 h after the treatment (Table 4).

**Table 4:** Aphicidal activity of five bacterial species cells on the pomegranate aphid, *A. punicae* under laboratory condition.

Bacterial species	Concentration (CFU mL^−1^)	^a^Mortality %				Bacterial species means
		6 h	12 h	24 h	48 h	
*S. tumulicola*	**10**^**2**^	^b^52 ± 4.9	64 ± 4	64 ± 4	72 ± 4.9	**82a**
	**10**^**4**^	60 ± 0	68 ± 4.9	76 ± 4	92 ± 4.9	
	**10**^**6**^	84 ± 4	88 ± 4.9	92 ± 4.9	100 ± 0	
	**10**^**8**^	100 ± 0	100 ± 0	100 ± 0	100 ± 0	
*P. saccharolyticum*	**10**^**2**^	16 ± 4	24 ± 4	36 ± 4	40 ± 6.3	**38.5d**
	**10**^**4**^	20 ± 0	36 ± 4	40 ± 0	44 ± 4	
	**10**^**6**^	24 ± 4	40 ± 0	44 ± 4	48 ± 4.9	
	**10**^**8**^	40 ± 0	48 ± 4.9	56 ± 4	60 ± 0	
*L. xylanilyticus* strain TU-2	**10**^**2**^	12 ± 4.9	20 ± 6.3	24 ± 4	32 ± 4.9	**44.8c**
	**10**^**4**^	28 ± 4.9	36 ± 4	40 ± 0	40 ± 0	
	**10**^**6**^	44 ± 4	48 ± 4.9	64 ± 4	64 ± 4	
	**10**^**8**^	48 ± 4.9	60 ± 0	72 ± 4.9	84 ± 4	
*S. liquefaciens*	**10**^**2**^	28 ± 4.9	32 ± 4.9	36 ± 4	52 ± 4.9	**49.8b**
	**10**^**4**^	28 ± 4.9	40 ± 0	44 ± 4	52 ± 4.9	
	**10**^**6**^	32 ± 4.9	44 ± 4	52 ± 4.9	72 ± 4.9	
	**10**^**8**^	48 ± 4.9	60 ± 0	80 ± 0	96 ± 4	
*L. xylanilyticus* strain BN-13	**10**^**2**^	4 ± 4	8 ± 4.9	12 ± 4.9	12 ± 4.9	**17.3e**
	**10**^**4**^	12 ± 4.9	12 ± 4.9	16 ± 4	16 ± 4	
	**10**^**6**^	12 ± 4.9	16 ± 4	20 ± 0	24 ± 4	
	**10**^**8**^	20 ± 0	20 ± 0	32 ± 4.9	40 ± 0	
Control		0 ± 0	0 ± 0	0 ± 0	0 ± 0	**0f**
Exposure time means		**29.7 d**	**36 c**	**41.7 b**	**47.5 a**	

a Five replicates of each treatment were used in this experiment, each with 20 adults insect.

b Numbers in each column indicated to mortality ± standard error.

Within the same column or row, means with different letters differ significantly (*p* < 0.05 using Duncan’s multiple range test).

As shown in Table 5, of all the tested bacterial species, *S. tumulicola* cells were the most effective in terms of toxicity against *A. punicae* adults 48 h after treatment, with an LC_50_ of 2.69 × 10^1^ CFU/ml and an LC_90_ of 1.55 × 10^3^ CFU/ml. In comparison, *S. liquefaciens* cells recorded an LC_50_ of 2.34 × 10^3^ and an LC_90_ of 1.75 × 10^7^ CFU/ml (Table 4), and *L. xylanilyticus* strain BN-13 cells recorded higher LC_50_ and LC_90_ values of 1.55 × 10^9^ and 1.35 × 10^18^ CFU/ml, respectively. It was also clear that *S. tumulicola* and *S. liquefaciens* isolates exhibited high slope values (3.68 and 2.56), which indicates homogeneity in the pomegranate aphid response to these bacteria (Table 4).

**Table 5:** Toxicity of five bacterial species cells against *A. punicae* after 48 h of exposure.

Bacterial species	LC_50_ CFU ml^−1^ (95% LCL–UCL)	LC_90_ CFU ml^−1^ (95% LCL–UCL)	Slope ± SE	Intercept	*X* ^2^	*p*-Value
*S. tumulicola*	2.69 × 10 (1.04–1.7)^*^	1.55 × 10^3^ (2.8–3.8)	3.68 ± 0.57	−0.57	3.40	0
*P. saccharolyticum*	1.62 × 10^6^ (3.9–8.4)	7.59 × 10^9^ (7.1–13.5)	0.75 ± 0.28	−0.53	1.87	0.008
*L. xylanilyticus* strain TU-2	2.51 × 10^4^ (3.8–4.9)	3.89 × 10^9^ (8.5–11.2)	2.27 ± 0.30	−1.33	2.98	0
*S. liquefaciens*	2.34 × 10^3^ (1.6–4.4)	1.75 × 10^7^ (6.4–10.4)	2.56 ± 0.69	−1.21	3.50	0
*L. xylanilyticus* strain BN-13	1.55 × 10^9^ (8.5–13.3)	1.35 × 10^15^ (14.4–17.9)	1.51 ± 0.34	−1.76	3.61	0

* Each figure represented as a power of 10.

LC_50_, lethal concentration that killing 50% of insects; LC_90_, lethal concentration that kills 90% of insects; LCL, lower confidence limit; UCL, upper confidence limit; *X*^2^, Chi-square value; SE, standard error; and *p*-value, probability.

#### Toxicity of bacterial filtrates to *Aphis illinoisensis* and *Aphis punicae*

The same tendency was observed when the influence of bacterial filtrate on the mortality of *A. illinoisensis* adults was examined (Table 6). According to these findings, individual mortality was also found to be highly influenced by bacterial species, filtrate concentration, and exposure time (*p* < 0.05). *S. tumulicola* exceeded all of the other tested bacteria in *A. illinoisensis* mortality at all concentrations and exposure periods tested. *S. tumulicola* induced an adult mortality rate of 73.5%, and isolates *S. liquefaciens*, *L. xylanilyticus* strain TU-2, *P. saccharolyticum* and *L. xylanilyticus* strain BN-13 induced mortality rates of 41.8, 36.8%, 30.5 and 9%, respectively. *A. illinoisensis* adults were significantly killed by a bacterial cell-free suspension at 600 μl/ml (46.2%; *p* < 0.05). Exposure time significantly affected the mortality rate (*p* < 0.05); a 41% mortality rate was observed 48 h post-exposure. As appeared in Table 2, aphid mortality (means ± SE) ranged from 44 to 100%, 20 to 88%, 4 to 76%, 8 to 52, and 0 to 32%, respectively (*p* < 0.05) when *S. tumulicola*, *S. liquefaciens*, *L. xylanilyticus* strain TU-2, *P. saccharolyticum* and *L. xylanilyticus* strain BN-13 were applied at 100–600 μl/ml. Furthermore, the cell-free supernatant of *L. xylanilyticus* strain BN-13 did not cause any mortality at 100 μl/ml at any tested times, and initiates toxicity (4% mortality) up to 32% when tested at 200–600 μl/ml (Table 6).

**Table 6:** Aphicidal activity of five bacterial species filtrates on the grapevine aphid, *A. illinoisensis* under laboratory condition.

Bacterial species	Concentration (μL mL^−1^)	^a^Mortality %				Bacterial species means
		6 h	12 h	24 h	48 h	
*S. tumulicola*	**100**	^b^44 ± 4	56 ± 4	56 ± 4	64 ± 4	**73.5a**
	**200**	52 ± 4.9	60 ± 0	68 ± 4.9	84 ± 4	
	**400**	68 ± 4.9	76 ± 4	84 ± 4	92 ± 4.9	
	**600**	80 ± 0	92 ± 4.9	100 ± 0	100 ± 0	
*P. saccharolyticum*	**100**	8 ± 4.9	16 ± 4	28 ± 4.9	32 ± 4.9	**30.5d**
	**200**	12 ± 4.9	28 ± 4.9	32 ± 4.9	36 ± 4	
	**400**	16 ± 4	32 ± 4.9	36 ± 4	40 ± 0	
	**600**	32 ± 4.9	40 ± 0	48 ± 4.9	52 ± 4.9	
*L. xylanilyticus* strain TU-2	**100**	4 ± 4	12 ± 8	16 ± 7.5	24 ± 4	**36.8c**
	**200**	20 ± 0	28 ± 4.9	32 ± 4.9	32 ± 4.9	
	**400**	36 ± 4	40 ± 0	56 ± 4	56 ± 4	
	**600**	40 ± 0	52 ± 4.9	64 ± 4	76 ± 4	
*S. liquefaciens*	**100**	20 ± 0	24 ± 4	28 ± 4.9	44 ± 4	**41.8b**
	**200**	20 ± 0	32 ± 4.9	36 ± 4	44 ± 4	
	**400**	24 ± 4	36 ± 7.5	44 ± 4	64 ± 4	
	**600**	40 ± 6.3	52 ± 4.9	72 ± 4.9	88 ± 4.8	
*L. xylanilyticus* strain BN-13	**100**	0 ± 0	0 ± 0	0 ± 0	0 ± 0	**9e**
	**200**	4 ± 4	4 ± 4	8 ± 4.9	8 ± 4.9	
	**400**	4 ± 4	8 ± 4.9	12 ± 4.9	16 ± 4	
	**600**	12 ± 4.9	12 ± 4.9	24 ± 4	32 ± 4.9	
Control		0 ± 0	0 ± 0	0 ± 0	0 ± 0	**0f**
Exposure time means		**22.3d**	**29.2c**	**35.2b**	**41a**	

a Each treatment was represented by five replicates, each with 20 adults insect.

b Mortality ± standard error is shown by the numbers in each column.

Means with different letters within the same column or row differ significantly (*p* < 0.05 using Duncan’s multiple range test).

Likewise, the data presented in Table 7 clarified that the filtrate of *S. tumulicola* isolate was more effective against grapevine aphid adults than the filtrates of all other tested isolates; the LC_50_ and LC_90_ at 48 h after treatment were 70.3 and 271.1 μl/ml, respectively. It’s also worth mentioning that *L. xylanilyticus* strain BN-13 and *P. saccharolyticum* isolates, having values of LC_50_ (958.8 and 586.5 μl/ml) and LC_90_ values of 3,318 and 1888 μl/ml, respectively, after 48 h were the least effective (Table 7). The highest degree of homogeneity for *A. illinoisensis* was observed for *S. tumulicola* (slope value of 2.38), and Low slope values were recorded for the other bacterial filtrate concentrations examined, indicating that the grapevine aphid response to these concentrations was heterogeneous (Table 7).

**Table 7:** Toxicity of five bacterial species filtrates against *A. illinoisensis* after 48 h of exposure.

Bacterial species	LC_50_ μl ml^−1^ (95% LCL–UCL)	LC_90_ μl ml^−1^ (95% LCL–UCL)	Slope ± SE	Intercept	*X^2^*	*p*-Value
*S. tumulicola*	70.3 (45.8–91.4)^*^	271.1 (225.3–350.1)	2.38 ± 0.40	−4.04	4.17	0
*P. saccharolyticum*	586.5 (440.3–1,129)	1888 (1261–4,929)	1.5 ± 0.023	−1.7	1.39	0.005
*L. xylanilyticus* strain TU-2	294.7 (249.5–351.9)	1,512 (1040–2763.1)	1.81 ± 0.23	−4.46	4.30	0
*S. liquefaciens*	212.9 (148.7–263.3)	700.6 (603.7–862.1)	2.19 ± 0.32	−3.35	4.23	0
*L. xylanilyticus* strain BN-13	958.8 (737.5–1,544)	3,318 (1924.3–9,550)	0.61 ± 0.22	−7.09	2.85	0

* Each figure represented as a power of 10.

LC_50_, lethal concentration that kills 50% of insects; LC_90_, lethal concentration that kills 90% of insects; LCL, lower confidence limit; UCL, upper confidence limit; *X*^2^, Chi-square value; SE, standard error; and *p*-value, probability.

The mortality rates of *A. punicae* were statistically significant (*p* < 0.05) at all concentrations from 6 to 48 h (Table 8). The data’s regression analysis revealed that mortality of *A. punicae* adults significantly increased with bacterial concentration (*R*^2^ = 0.938; *p* < 0.05). When they were treated with 600 and 100 μl/ml, respectively, they had the highest (49.3%) and lowest (23.3%) mortality rates. Among all tested bacterial species, *S. tumulicola* was the most toxic: it induced a 77.3% mortality rate in *A. punicae* adults, whereas *S. liquefaciens*, *L. xylanilyticus* strain TU-2, *P. saccharolyticum* and *L. xylanilyticus* strain BN-13 isolates induced mortality rates of 45.8, 40.8, 34.5 and 13.3%, respectively. The overall mortality of the *A. punicae* adults after treatment with 100–600 μl/ml of *S. tumulicola* filtrate ranged from 48 to 100%, while it ranged from 24 to 92% for *S. liquefaciens*, and from 8 to 80% for *L. xylanilyticus* strain TU-2. In contrast, it ranged from 0 and 36% and from 12 to 56% for *L. xylanilyticus* strain BN-13 and *P. saccharolyticum*, respectively. Table 8 shows that the *A. punicae* adults were extremely vulnerable (*p* < 0.05) to both the *S. tumulicola* and *S. liquefaciens* filtrates, since they recorded 100 and 92% mortality 48 h post treatment. *S. tumulicola* induced 100% individual mortality at 24 and 48 h post-treatment, and *S. liquefaciens* induced 76 and 92% mortality at 600 μl/ml at the same exposure times, respectively. There was also an increase in adult mortality as the bacterial filtrate concentration and exposure time increased (Table 8).

**Table 8:** Aphicidal activity of five bacterial species filtrates on the pomegranate aphid, *A. punicae* under laboratory condition.

Bacterial species	Concentration (μL ml^−1^)	^a^Mortality %				Bacterial species Means
		6 h	12 h	24 h	48 h	
*S. tumulicola*	**100**	^**b**^48 ± 4.9	60 ± 0	60 ± 0	68 ± 4.9	**77.3 a**
	**200**	56 ± 4	64 ± 4	72 ± 4.9	88 ± 4.9	
	**400**	72 ± 4.9	80 ± 0	88 ± 4.9	96 ± 4	
	**600**	88 ± 4.9	96 ± 4	100 ± 0	100 ± 0	
*P. saccharolyticum*	**100**	12 ± 4.9	20 ± 0	32 ± 4.9	36 ± 4	**34.5 d**
	**200**	16 ± 4	32 ± 4.9	36 ± 4	40 ± 0	
	**400**	20 ± 0	36 ± 4	40 ± 0	44 ± 4	
	**600**	36 ± 4	44 ± 4	52 ± 4.9	56 ± 4	
*L. xylanilyticus* strain TU-2	**100**	8 ± 4.9	16 ± 7.5	20 ± 6.3	28 ± 4.9	**40.8 c**
	**200**	24 ± 4	32 ± 4.9	36 ± 4	36 ± 4	
	**400**	40 ± 0	44 ± 4	60 ± 0	60 ± 0	
	**600**	44 ± 4	56 ± 4	68 ± 4.9	80 ± 0	
*S. liquefaciens*	**100**	24 ± 4	28 ± 4.9	32 ± 4.9	48 ± 4.9	**45.8 b**
	**200**	24 ± 4	36 ± 4	40 ± 0	48 ± 4.9	
	**400**	28 ± 4.9	40 ± 6.3	48 ± 4.9	68 ± 4.9	
	**600**	44 ± 4	56 ± 4	76 ± 4	92 ± 4.9	
*L. xylanilyticus* strain BN-13	**100**	0 ± 0	4 ± 4	8 ± 4.9	8 ± 4.9	**13.3 e**
	**200**	8 ± 4.9	8 ± 4.9	12 ± 4.9	12 ± 4.9	
	**400**	8 ± 4.9	12 ± 4.9	16 ± 4	20 ± 0	
	**600**	16 ± 4	16 ± 4	28 ± 4.9	36 ± 4	
Control		0 ± 0	0 ± 0	0 ± 0	0 ± 0	**0 f**
Exposure time means		**25.7 d**	**32.5 c**	**38.5 b**	**44.3 a**	

a Each treatment was represented by five replicates, each with 20 adults insect.^b^Numbers in each column indicates to mortality ± standard error.Means with different letters within the same column or row differ significantly (*p* < 0.05 using Duncan’s multiple range test).

The LC_50_ and LC_90_ values for each isolate at 48 h after treatment against *A. punicae* adults are shown in Table 9. The LC_50_ and LC_90_ values for *S. tumulicola* were 65.4 and 218.6 μl/ml. The LC_50_ and LC_90_ values for isolates *S. liquefaciens*, *L. xylanilyticus* strain TU-2, *P. saccharolyticum* and *L. xylanilyticus* strain BN-13 were 212.9 and 587.5 μl/ml, 312.1 and 755.3 μl/ml, 483.4 and 1,406.2 μl/ml, and 784.2 and 1,808.3 μl/ml, respectively. In addition, the *A. punicae* individuals exhibited different degrees of homogeneity in response to the tested bacterial filtrates. The slope values ranged from 0.59 to 2.45 (Table 9). Additionally, *S. tumulicola* had the highest degree of homogeneity for *A. punicae*, with a slope value of 2.45.

**Table 9:** Toxicity of five bacterial species filtrates against *A. punicae* 48 h post-exposure.

Bacterial species	LC_50_ μl ml^−1^ (95% LCL–UCL)	LC_90_ μl ml^−1^ (95% LCL–UCL)	Slope ± SE	Intercept	*X^2^*	*p*-Value
*S. tumulicola*	65.4 (42.2–84.8)	218.6 (183.9–274.6)	2.45 ± 0.37	−4.44	1.58	0
*P. saccharolyticum*	483.4 (353.9–864.3)	1406.2 (1119.6–2027)	0.59 ± 0.21	−1.59	1.41	0.006
*L. xylanilyticus* strain TU-2	312.1 (265.6–358.1)	755.3 (660.6–9.4.8)	1.79 ± 0.23	−4.31	5.02	0
*S. liquefaciens*	212.9 (184.2–229.1)	587.5 (516.7–696)	1.85 ± 0.24	−4.06	1.47	0
*L. xylanilyticus* strain BN-13	784.2 (657.2–1,043)	1808.3 (1206.8–4,806)	1.35 ± 0.27	−4.21	2.16	0

* Each figure represented as a power of 10.

LC_50_, lethal concentration that kills 50% of insects; LC_90_, lethal concentration that kills 90% of insects; LCL, lower confidence limit; UCL, upper confidence limit; *X*^2^, Chi-square value; SE, standard error; and *p*-value, probability.

## Discussion

The first phase of this research resulted in the successful recovery and isolation of EPB from *S. feltiae*. Five bacterial isolates associated with EPN *S. feltiae* were identified. These findings are consistent with and add to those previously reported by (Alotaibi et al., 2021 and Noureldeen et al., 2022), who isolated *Xenorhabdus* sp. and *Photorhabdus* sp. from the EPNs *Steinernema* sp. and *Heterorhabditis* sp., respectively, in the same region and recorded their complex activities against *Meloidogyne incognita*, which infects pomegranate under greenhouse conditions. The five isolated species of *Steinernema*-associated bacteria found here were molecularly identified and termed isolates; based on phylogenetic tree analysis, *L. xylanilyticus* strain TU-2, *L. xylanilyticus* strain BN-13, *S. tumulicola*, *S. liquefaciens* and *P. saccharolyticum*. In the present study, we found that these five isolates were symbiotically associated with *S. feltiae*, which were discovered for the first time in Saudi Arabia. The obtained data are in agreement with those earlier stated by (Ogier et al., (2020), on four species of *Steinernema* and their associated bacteria. In that study, two species, *Pseudomonas chlororaphis* and *Pseudomonas protegens*, which are often members of the associated microbiota possibly, engaged in *Steinernema*’s parasitic lifecycle, exhibited entomopathogenic capabilities, implying a role in the virulence and pathobiome membership of *Steinernema*. Also our results are confirmed by (Moawad and Al-Barty, (2011), Dirksen et al., (2016), Hartman et al., (2017), and Lacerda Júnior et al., (2017).

Regarding the insecticidal activity, it was clear that the five bacterial isolates were more effective on the pomegranate aphid under laboratory conditions than on the grapevine aphid, which showed low susceptibility. It was also clear that *S. tumulicola* and *S. liquefaciens* could control the pomegranate and grapevine aphids. The obtained results also accomplished that *S. tumulicola* isolate was better than *S. liquefaciens* against both *A. punicae* and *A. illinoisensis* adults; however, *A. illinoisensis* was more resistant. *S. tumulicola* isolate either cells or filtrates, showed aphicidal activity against *A. punicae* and *A. illinoisensis* systematically or through direct contact, causing lethality to the adult stage of both aphid species, and the accumulative mortality approached 100% 48 h following treatment. These results were in accordance with those of (Alotaibi et al., 2021) who observed that the two *Xenorhabdus* bacterial species EMA and EMC causing significant higher *E. ceratoniae* larvae mortality than that caused by the *Photorhabdus* species TT01. In a recent study conducted by Elbrense et al., (2021), it was discovered that *H. bacteriophora* and its symbiont, *Photorhabdus* sp., were more virulent against *Pieris rapae* and *Pentodon algerinus*. Our findings are in conformity with the results of (Jabeen et al., 2018), who measured the amount of bacterial chitinases produced by *S. maltophilia* and their termiticidal potential, and (Amer et al., 2021), who claimed that *S. maltophilia* had antimicrobial activity against a variety of multidrug-resistant bacteria and fungi. Likewise, *Rhizoctonia solani*, a phytopathogen, was inhibited by *S. maltophilia*, probably due to antibiosis and the production of lytic enzymes that eliminate fungi (Berg, 1996). Following research, it was shown that the synthesis of novel bioactive compounds is mostly due to *S. maltophilia*’s metabolic diversity, including molecules that could be employed in bio-control against microbes and insects (Ribitsch et al., 2012). Overall, bacterial isolates are showing more toxicity toward the aphids than the bacterial cell free extract. This is most likely because during the extraction process, significant metabolites of bacterial crude cell extract were lost, or they were never present. Second, various EPB species have variable protein densities, which is why different EPB results have varied. It has been established that the protein encoding density of several *Xenorhabdus* species varies greatly (Kim et al., 2017) While the crude cell extract was shown to be less effective than other fractions of EPB, the data clearly show that it contains certain harmful proteins that remain there after centrifugation and are responsible for death. However, the relationship between biological processes and temperature is also real and significant.

## Conclusion

In current study, we found that these five bacteria isolates were associated with *S. feltiae*, and be identified as the first recorded at Taif, Saudi Arabia. The effectiveness of these bacteria was examined against the two important insect pests of pomegranate and grapevine, *A. punicae* and *A. illinoisensis*. Our results offer a reliable base for promising biocontrol methods and agents that could be used in managing piercing-sucking insects. Further investigations are needed, especially regarding other associated microorganisms for developing new environmentally friendly insect pests control toward suitable agricultural production. In the future, the inoculation of these bacteria can be used directly as biocontrol agents or they can be used in combination with other available methods of biocontrol for better managements of insect pests.

## Data availability statement

The data presented in the study are deposited in the NCBI repository, accession number OP001649, OP002058, OP002063, OP578131, and OP578132.

## Author contributions

SSA and AN: conceptualization and writing—original draft. HD, SSA, and AN: data curation. HD, AN, SJ and AA-B: formal analysis. SSA, AA, and AN: investigation. HD, AN, MZ, and AA-B: methodology. SSA: project administration. SSA, SA, BA, and AN: resources. HD and AA: validation. AN and BA: visualization. SSA, LK, AB, UK, and AN: writing—review and editing. All authors contributed to the article and approved the submitted version.

## Funding

The current work was funded by Taif University Researchers Supporting Project number (TURSP-2020/295), Taif university, Taif, Saudi Arabia.

## Conflict of interest

The authors declare that the research was conducted in the absence of any commercial or financial relationships that could be construed as a potential conflict of interest.

## Publisher’s note

All claims expressed in this article are solely those of the authors and do not necessarily represent those of their affiliated organizations, or those of the publisher, the editors and the reviewers. Any product that may be evaluated in this article, or claim that may be made by its manufacturer, is not guaranteed or endorsed by the publisher.
